# Supplementary material for: A single Gal4-like transcription factor activates the Crabtree effect in Komagataella phaffii
Source: Nat Commun. 2018 Nov 21;9:4911. doi: 10.1038/s41467-018-07430-4 (PMC6249229; doi:10.1038/s41467-018-07430-4)
Supplement: Supplementary file 1 — Supplementary Information [file 41467_2018_7430_MOESM1_ESM.pdf]

Supplementary Information

A single Gal4-like transcription factor activates the Crabtree effect in *Komagataella phaffii*

Özge Ata, Corinna Rebnegger, Nadine E. Tatto, Minoska Valli, Teresa Mairinger, Stephan Hann, Matthias G. Steiger, Pınar Çalık, Diethard Mattanovich

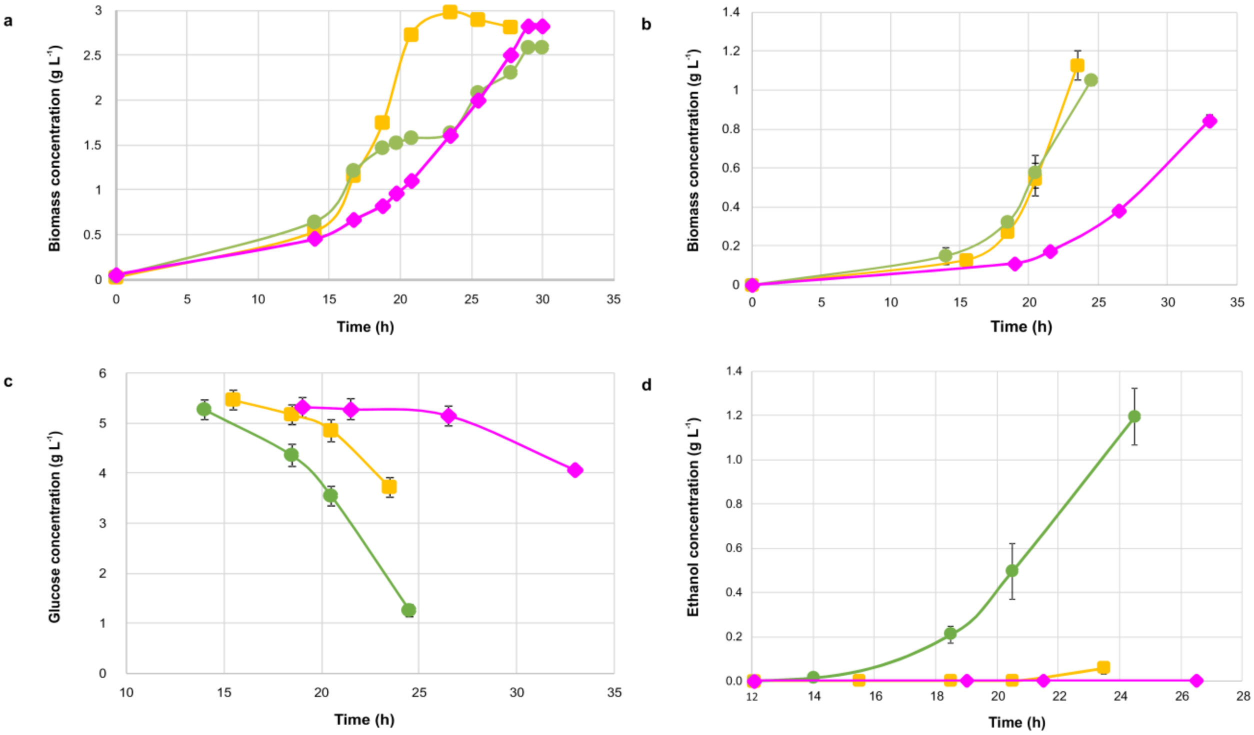

Supplementary Figure 1. Profiles of biomass dry weight, glucose consumption and ethanol formation in batch cultures. Control (yellow line-square), *CRA1* (= *PpGAL4*) overexpression (green line-circle), *Δcra1* strains (magenta line-diamond), harbouring eGFP gene under control of the GAP promoter as a reporter of *CRA1* based induction of glycolytic genes. **(a)** biomass dry weight of the strains in batch cultivation until stationary phase is reached. The Cra1 overexpression strain showed a diauxic shift typical for a Crabtree positive yeast. **(b)** biomass dry weight during the exponential phase. **(c)** glucose concentration during the exponential phase. **(d)** ethanol production during the exponential phase. Cells were grown on glucose (5 g L^{-1}) in shake flask and samples were collected during the exponential phase. Error bars show the standard deviation (±) of duplicate experiments.

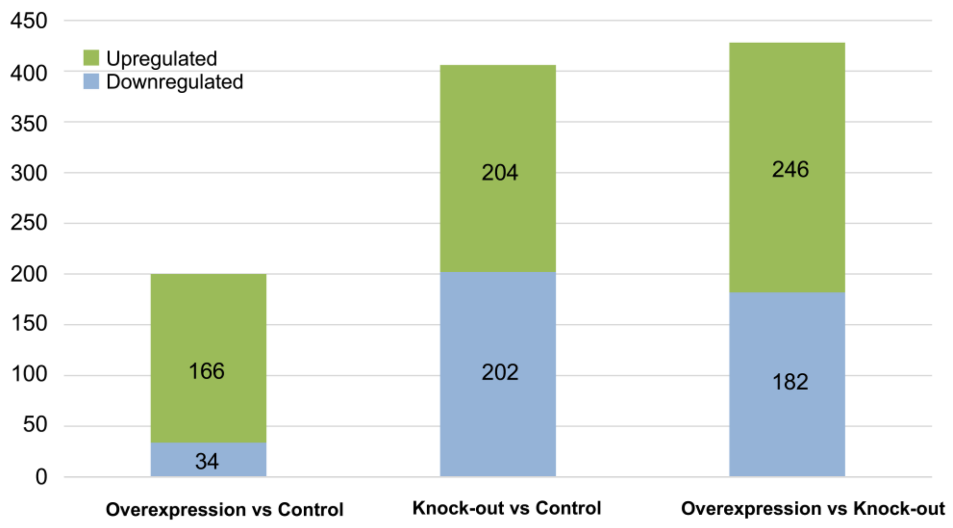

Supplementary Figure 2. Summary of the total number of regulated genes of each comparison in the RNASeq data.

Supplementary Table 1. **Analysis of short-term Crabtree phenotype.**

Strain	Time [min]	Biomass [g L^{-1}]	q_S[a] [C-mmol g^{-1} h^{-1}]	q_{EtOH}[b] [C-mmol g^{-1} h^{-1}]	OUR[c] [mmol g^{-1} h^{-1}]	CER[d] [C-mmol g^{-1} h^{-1}]
**Control**	SS[e]	3.68±0.00	6.36±0.14	0	1.81±0.01	1.48±0.07
	0	3.68±0.00			1.80±0.01	1.48±0.07
	20	3.67±0.24	19.09±0.29	0	2.17±0.24	2.64±0.22
	40	3.92±0.17	15.60±2.37	0	2.16±0.19	2.57±0.21
	60	4.63±0.10	15.79±6.62	0	1.81±0.09	2.17±0.14
	80	4.74±0.14	6.07±6.42	0	2.33±0.44	2.10±0.10
	100	4.24±0.03	12.15±0.54	0	2.56±0.37	2.35±0.02
	120	4.73±0.04	11.23±1.17	0	2.28±0.33	2.10±0.05
***CRA1* overexpression**	SS	3.64±0.08	6.62±0.01	0	1.69±0.34	1.48±0.10
	0	3.6±0.20			1.69±0.36	1.47±0.08
	20	3.42±0.20	23.08±1.14	2.02±0.48	2.41±0.68	4.53±0.02
	40	3.47±0.21	19.93±2.62	0.63±0.42	2.21±0.50	3.36±0.14
	60	3.68±0.34	16.21±3.62	0.65±0.24	2.76±0.37	3.12±0.09
	80	4.29±0.52	11.92±1.27	0.036±0.04	2.47±0.41	2.64±0.03
	100	4.53±0.10	10.78±0.06	-0.39±0.54	2.58±0.39	2.61±0.01
	120	4.30±0.62	11.62±0.81	-1.69±0.99	2.47±0.31	2.31±0.05

[a] Specific glucose consumption rates
[b] Specific ethanol production rates
[c] Oxygen uptake rates
[d] Carbon dioxide evolution rates
[e] SS refers to steady-state (D = h^{-1}) before the glucose pulse was applied. ± indicates the standard deviation of duplicate experiments.

Supplementary Table 2. **Analysis of long-term Crabtree phenotype [a].**

Strain	D[b] [h^{-1}]	Biomass [g L^{-1}]	q_S[c] [C-mmol g^{-1} h^{-1}]	q_{EtOH}[d] [C-mmol g^{-1} h^{-1}]	OUR[e] [mmol g^{-1} h^{-1}]	CER[f] [C-mmol g^{-1} h^{-1}]	$Y_{X/S}$[g] [g g^{-1}]
**Control**	0.1	6.11±0.13	5.22±0.21	0	0.74±0.01	0.72±0.03	0.61±0.01
	0.15	6.4±0.03		0	0.95±0.00	0.95±0.04	0.64±0.00
	0.2	6.11±0.13	10.44±0.42	0	1.24±0.05	1.35±0.01	0.61±0.01
	0.225	5.84±0.17	12.28±0.13	0	1.47±0.01	1.58±0.05	0.58±0.02
	0.275	5.8±0.06	15.13±0.46	0	1.70±0.03	1.81±0.01	0.58±0.01
	0.3	6.14±0.03	15.58±0.24	0	2.12±0.05	2.28±0.12	0.55±0.02
	0.4	5.73±0.30	22.28±0.73	0	2.18±0.34	2.31±0.45	0.57±0.03
***CRA1* overexpression**	0.1	6.32±0.34	4.74±0.03	0	0.90±0.05	0.72±0.01	0.63±0.03
	0.15	6.2±0.08	7.25±0.24	0	1.00±0.27	1.03±0.00	0.62±0.01
	0.2	6.32±0.34	9.49±0.07	0	1.24±0.26	1.38±0.04	0.63±0.03
	0.225	5.81±0.16	11.6±0.23	0	1.49±0.34	1.68±0.02	0.58±0.02
	0.275	5.24±0.62	15.8±1.15	0	1.88±0.14	2.11±0.21	0.52±0.06
	0.3	2.36±0.62	33.14±4.08	13.47±2.66	2.63±0.48	3.94±0.87	0.27±0.02

[a] ± indicates the standard deviation of duplicate experiments. [b] Dilution rate
[c] Specific glucose consumption rate
[d] Specific ethanol production rate
[e] Oxygen uptake rate
[f] Carbon dioxide evolution rate
[g] Yield of biomass on substrate

Supplementary Table 3. Analysis of the glycolytic and galactose metabolism promoters of different yeast species.

	POST WGD			PRE-WGD									
**Species** / **Promoter[a]**	*S. cerevisiae* +	*K. naganishii* +	*N. castellii* +	*Z. rouxii* +	*L. kluyveri* +	*E. gossypii*	*K. lactis* +	*C. albicans* +	*S. lignohabitans* +	*D. hansenii* +	*Y. lipolytica* *	*O. parapolymorpha*	*K. phaffii*
Hexokinase	2	2	2	2	4	0	0	0	0	2	0	6	4
Phosphoglucose isomerase	0	2	2	0	0	0	0	0	4	2	2	2	2
Phosphofructokinase	0	8	0	0	0	8	0	2	0	4	2	4	2
Fructose 1,6-bisphosphate aldolase	0	0	0	0	0	2	0	2	0	0	0	0	4
Fructose-1,6-bisphosphatase	0	0	0	0	0	2	2	0	2	0	0	2	2
Triose phosphate isomerase	0	0	0	0	0	0	0	2	4	2	0	2	4
Glyceraldehyde-3-phosphate dehydrogenase	0	8	0	0	0	0	0	2	4	2	2	2	2
3-phosphoglycerate kinase	0	2	2	4	0	2	0	0	2	2	0	2	2
Phosphoglycerate mutase	0	0	0	0	0	2	0	2	0	2	0	4	2
Enolase	2	0	2	0	0	2	0	0	0	2	2	4	0
Pyruvate kinase	2	0	0	4	0	0	0	2	2	2	0	4	4
Pyruvate decarboxylase	0	2	2	0	0	2	0	0	4	0	0	0	2
Alcohol dehydrogenase	0	2	0	6	0	2	4	6	0	2	0	8	2
Galactokinase	8	10	14	4	6	-	8	0	0	0	0	-	-
Galactose-1-phosphate uridyl transferase	6	4	16	6	6	-	4	0	0	0	0	-	-
UDP-glucose-4-epimerase	10	8	6	4	4	-	8	0	2	0	2	-	-
**Total (Glycolytic)**	6	26	10	16	4	22	6	18	22	22	8	38	32
**Total (Galactolytic)**	24	22	36	14	16	-	20	0	2	0	2	-	-

[a]Promoter sequences of *Saccharomyces cerevisiae* (S288c), *Kazachstania naganishii* (CBS8797), *Naumovozyma castellii* (CBS4309) (syn. *Saccharomyces castellii*), *Zygosaccharomyces rouxii* (CBS732), *Lachancea kluyveri* (CBS3082) (syn. *Saccharomyces kluyveri*), *Eremothecium gossypii* (ATCC10895) (syn. *Ashbya gossypii*), *Kluyveromyces lactis* (CBS2359), *Candida albicans* (SC5314), *Sugiyamaella lignohabitans* (CBS10342), *Debaryomyces hansenii* (CBS767), *Yarrowia lipolytica* (CLIB122), and *Komagataella phaffii* (CBS7435) were analyzed for the presence of the putative Gal4 binding sites. Putative TFBSs were searched by using MatInspector[1]. Only matches with a predicted binding site on both DNA strands were considered as true binding sites. For genes that have multiple subunits or homologs, total number of the binding sites is given. Total numbers of the putative Gal4 binding sites on the glycolytic or galactolytic promoters are shown. ("+" can utilize galactose, "*" has the galactose metabolism genes but they are not active).

Supplementary Table 4. Growth characteristics of the control and *GAL4* overexpression strain of *Sugiyamaella lignohabitans*[a].

	μ[b] [h^{-1}]	q_s[c] [$g\ g^{-1}\ h^{-1}$]	q_{Gly}[d] [$g\ g^{-1}\ h^{-1}$]	$Y_{X/S}$[e] [$g\ g^{-1}$]
S. lignohabitans control	0.30±0.01	0.76±0.00	0.0011±0.0001	0.40±0.02
S. lignohabitans GAL4 overexpression	0.35±0.01	0.99±0.11	0.0016±0.0001	0.35±0.02

[a]Average values of two independent cultures for the control and the five randomly selected transformants are given. ± indicates the standard deviation
[b] Specific growth rate
[c] Specific glucose consumption rate
[d] Specific glycerol production rate
[e] Yield of biomass on substrate
.

Supplementary Table 5. Stoichiometric model for the ^{13}C flux measurements in OpenFLUX based on the model published by Baumann *et al*. 2010[2].

RxnID	Reaction Equation	Carbon transition	Openflux Rxn Type
**R1**	MeOH_EX = MeOHint	a = a	F
**R2**	MeOHint = FORM	a = a	F
**R3**	FORM = FOR + NADH	a = a + X	F
**R4**	FOR = CO2 + NADH	a = a + X	F
**R5**	XYL5P + FORM = DHA + G3P	abcde + f = fab + cde	F
**R6**	DHA = DHAP	abc = abc	FR
**R7**	DHAP = DHA	abc = abc	R
**R8**	DHA = GLYO	abc = abc	F
**R9**	GLC_EX + ATP = G6P + ADP	abcdef + X = abcdef + X	F
**R10**	G6P = F6P	abcdef = abcdef	F
**R11**	F6P + ATP = FBP + ADP	abcdef + X = abcdef + X	F
**R12**	FBP = F6P + Pi	abcdef = abcdef + X	F
**R13**	FBP = G3P + G3P	abcdef = abc + def	F
**R14**	G3P + ADP + NAD = PG3 + ATP + NADH	abc + X + X = abc + X + X	F
**R15**	PG3 = PEP	abc = abc	F
**R16**	PEP + ADP = PYR + ATP	abc + X = abc + X	F
**R17**	PYR + NAD = ACCoA + CO2 + NADH	abc + X = bc + a + X	F
**R18**	PYR + CO2 + ADP = OAA + ATP	abc + d + X = abcd + X	F
**R19**	PYR = AcO + CO2	abc = bc + a	F
**R20**	G6P + NADP = RUL5P + CO2 + NADPH	abcdef + X = bcdef + a + X	F
**R21**	RUL5P = XYL5P	abcde = abcde	F
**R22**	XYL5P + ADP = XYL + ATP	abcde + X = abcde + X	F
**R23**	XYL + NADH = AROL + NAD	abcde + X = abcde + X	F
**R24**	RUL5P = RIB5P	abcde = abcde	F
**R25**	XYL5P + RIB5P = SED7P + G3P	abcde + fghij = fgabcde + hij	FR
**R26**	SED7P + G3P = XYL5P + RIB5P	abcdefg + hij = cdefg + abhij	R
**R27**	SED7P + G3P = E4P + F6P	abcdefg + hij = defg + abchij	FR
**R28**	E4P + F6P = SED7P + G3P	abcd + efghij = efgabcd + hij	R
**R29**	XYL5P + E4P = F6P + G3P	abcde + fghi = abfghi + cde	FR
**R30**	F6P + G3P =XYL5P + E4P	abcdef + ghi = abghi + cdef	R
**R31**	ACCoA + OAA = CIT	ab + cdef = fedbac	F
**R32**	CIT = ICIT	abcdef = abcdef	F
**R33**	ICIT + NAD = AKG + CO2 + NADH	abcdef + X = abcde + f + X	F
**R34**	AKG + NAD + ADP + FAD = 0.5 SUCC + 0.5 SUCC + CO2 + NADH + FADH2 + ATP	abcde + X + X + X = 0.5 bcde + 0.5 edcb + a + X + X + X	F
**R35**	SUCC + NAD = FUM + NADH	abcd + X = abcd + X	F
**R36**	FUM + H2O = MAL	abcd + X = abcd	F

**R37**	MAL + NAD = OAA + NADH	abcd + X = abcd + X	FR
**R38**	OAA + NADH = MAL + NAD	abcd + X = abcd + X	R
**R39**	ICIT = GLYOXY + 0.5 SUCC + 0.5 SUCC	abcdef = ab + 0.5 fcde + 0.5 edcf	F
**R40**	ACCoA + GLYOXY = MAL	ab + cd = cdba	F
**R41**	AcO + NADH = ETOH + NAD	ab + X = ab + X	F
**R42**	AcO + ATP = Ac + ADP	ab + X = ab + X	F
**R43**	AcO + NADP + CoA + ATP = ACCoA + NADPH + Pi + AMP	ab + X + X + X = ab + X + X + X	F
**R44**	G3P + NADH + ATP = GLYO + Pi + ADP + NAD	abc + X + X = abc + X + X + X	FR
**R45**	GLYO + Pi + ADP + NAD = G3P + ATP + NADH	abc + X + X + X = abc + X + X	R
**R46**	GLYO = GLYOex		B
**R47**	PYR = PYRex		B
**R48**	CIT = CITex		B
**R49**	CO2 = CO2ex		B
**R50**	AcO = AcOex		B
**R51**	Ac = Acex		B
**R52**	ETOH = ETOHex		B
**R53**	AROL = AROLex		B
**R54**	0.136 PYR + 0.006 RIB5P + 0.013 E4P + 0.040 OAA + 0.075 AKG + 0.027 ACCoA = Protein + 0.002 G3P + 0.058 CO2		B
**R55**	0.113 G6P + 0.053 F6P + 0.167 ATP = Carbohydrate + 0.167 ADP		B
**R56**	0.002 G6P + 0.0055 PYR + 0.011 G3P + 0.006 CO2 + 0.039 ACCoA + 0.441 ACCoA + 0.07 NADH + 0.599 NADPH + 0.42 ATP + 0.065 O2 = Lipid + 0.07 NAD + 0.599 NADP + 0.42 ADP + 0.42 Pi		B
**R57**	0.056 PYR + 0.1136 CO2 + 0.105 RIB5P + 0.104 NAD + 0.075 NADPH + 1.1 ATP + 0.0479 OAA = RNA + 1.1 Pi + 1.1 ADP + 0.075 NADP + 0.104 NADH		B
**R58**	0.051 PYR + 0.132 CO2 + 0.102 RIB5P + 0.102 NAD + 0.273 NADPH + 1.146 ATP + 0.051 OAA = DNA + 1.146 Pi + 1.146 ADP + 0.273 NADP + 0.102 NADH		B
**R59**	0.455286007065888 Protein + 0.0546300247279858 RNA + 0.00113541838465316 DNA + 0.106302993687774 Lipid + 0.382645556133698 Carbohydrate + ATP = Biomass + ADP + Pi		B
**R60**	PYR = ALA	abc = abc	S
**R61**	OAA = ASP	abcd = abcd	S
**R62**	OAA = ASN	abcd = abcd	S
**R63**	AKG = GLU	abcde = abcde	S
**R64**	AKG = GLN	abcde = abcde	S
**R65**	G3P = 3PG	abc = abc	S
**R66**	3PG = SER	abc = abc	S
**R67**	SER = GLY + MTHF	abc = ab + c	S
**R68**	OAA = THR	abcd = abcd	S
**R69**	THR = GLY + ACETAL	abcd = ab + cd	S
**R70**	PYR + PYR = VAL + CO2	abc + def = abefc + d	S
**R71**	E4P + PYR = SHKM	abcd + efg = efgabcd	S
**R72**	SHKM + PYR = CHRM	abcdefg + hij = abcdefghij	S

**R73**	CHRM = PHE + CO2	abcdefghij = hijbcdefg + a	S
**R74**	CHRM = TYR + CO2	abcdefghij = hijbcdefg + a	S
**R75**	CHRM = ANTHR + PYR	abcdefghij = abcdefg + hij	S
**R76**	ANTHR + RIB5P = CPADRIB5P	abcdefg + hijkl = abcdefghijkl	S
**R77**	CPADRIB5P = INDG + CO2	abcdefghijkl = abcdfghijkl + e	S
**R78**	INDG = IND + G3P	abcdefghijk = abcdefgh + ijk	S
**R79**	IND + 3PG = TRP	abcdefgh + ijk = abcdefghkji	S
**R80**	PYR + OAA = ILE + CO2	abc + defg = debfgc + a	S
**R81**	PYR + PYR = ISV + CO2	abc + def = abefc + d	S
**R82**	ISV + ACCoA = LEU + CO2	abcde + fg = fgbcde + a	S
**R83**	AKG + CO2 = ARG	abcde + f = abcdef	S
**R84**	3PG = CYS	abc = abc	S
**R85**	OAA + MTHF = MET	abcd + e = abcde	S
**R86**	AKG = PRO	abcde = abcde	S
**R87**	AKG + ACCoA = LYS + CO2	abcde + fg = fgbcde + a	S
**R88**	RIB5P + MTHF = HIS	abcde + f = edcbaf	S

Supplementary Table 6. Mass distribution values (MDV) of the control, *CRA1* overexpression and knock-out strains and the respective deviations of 3 biological replicates.

Metabolite[a]	Control			*CRA1* overexpression			*CRA1* knock-out		
	MDV	Dev (±)	Sim[b]	MDV	Dev (±)	Sim	MDV	Dev (±)	Sim
GAP_M	0.154	0.008	0.127	0.094	0.007	0.047	0.151	0.007	0.093
GAP_M1	0.748	0.007	0.854	0.802	0.021	0.927	0.779	0.012	0.870
GAP_M2	0.090	0.009	0.018	0.097	0.013	0.027	0.062	0.009	0.037
GAP_M3	0.007	0.006	0.000	0.007	0.006	0.000	0.008	0.001	0.000
R5P_M	0.052	0.001	0.039	0.036	0.001	0.011	0.091	0.002	0.034
R5P_M1	0.849	0.005	0.921	0.833	0.014	0.931	0.784	0.013	0.895
R5P_M2	0.099	0.004	0.040	0.130	0.014	0.057	0.124	0.012	0.068
R5P_M3	0.000	0.000	0.001	0.001	0.001	0.001	0.002	0.003	0.002
R5P_M4	0.000	0.000	0.000	0.000	0.000	0.000	0.000	0.000	0.000
R5P_M5	0.000	0.000	0.000	0.000	0.000	0.000	0.000	0.000	0.000
F6P_M	0.045	0.000	0.045	0.014	0.003	0.001	0.048	0.002	0.025
F6P_M1	0.338	0.013	0.381	0.164	0.005	0.099	0.370	0.009	0.293
F6P_M2	0.560	0.011	0.550	0.780	0.018	0.848	0.537	0.004	0.594
F6P_M3	0.020	0.029	0.023	0.004	0.005	0.051	0.002	0.004	0.085
F6P_M4	0.032	0.024	0.000	0.036	0.015	0.001	0.043	0.005	0.003
F6P_M5	0.004	0.000	0.000	0.002	0.003	0.000	0.000	0.000	0.000
F6P_M6	0.000	0.000	0.000	0.001	0.001	0.000	0.000	0.000	0.000
G6P_M	0.015	0.001	0.012	0.006	0.000	0.001	0.015	0.000	0.008
G6P_M1	0.152	0.002	0.112	0.116	0.001	0.099	0.119	0.002	0.110
G6P_M2	0.783	0.008	0.840	0.825	0.002	0.848	0.811	0.001	0.824
G6P_M3	0.015	0.005	0.036	0.019	0.003	0.051	0.017	0.002	0.055
G6P_M4	0.035	0.007	0.001	0.034	0.004	0.001	0.039	0.004	0.002
G6P_M5	0.000	0.000	0.000	0.000	0.000	0.000	0.000	0.000	0.000
G6P_M6	0.000	0.000	0.000	0.000	0.000	0.000	0.000	0.000	0.000
3PG_M0	0.127	0.004	0.127	0.069	0.004	0.047	0.150	0.002	0.093
3PG_M1	0.870	0.005	0.854	0.923	0.005	0.927	0.821	0.003	0.870
3PG_M2	0.004	0.006	0.018	0.007	0.004	0.027	0.030	0.001	0.037
3PG_M3	0.000	0.000	0.000	0.000	0.000	0.000	0.000	0.000	0.000
AKG_M0	0.025	0.001	0.041	0.024	0.001	0.080	0.035	0.002	0.040
AKG_M1	0.150	0.001	0.226	0.133	0.002	0.310	0.169	0.002	0.231
AKG_M2	0.470	0.003	0.422	0.604	0.005	0.408	0.491	0.003	0.438
AKG_M3	0.308	0.001	0.283	0.221	0.002	0.190	0.273	0.000	0.273
AKG_M4	0.048	0.002	0.029	0.018	0.001	0.011	0.032	0.002	0.018
AKG_M5	0.000	0.000	0.000	0.000	0.000	0.000	0.000	0.000	0.000
Fum_M0	0.050	0.000	0.088	0.049	0.001	0.160	0.062	0.001	0.100
Fum_M1	0.364	0.002	0.356	0.595	0.004	0.440	0.406	0.001	0.396
Fum_M2	0.463	0.003	0.426	0.323	0.003	0.344	0.433	0.001	0.420
Fum_M3	0.123	0.001	0.129	0.033	0.002	0.055	0.098	0.001	0.084

**Fum_M4**	0.000	0.000	0.001	0.000	0.000	0.001	0.001	0.001	0.001
**Mal_M0**	0.113	0.001	0.096	0.147	0.002	0.170	0.134	0.006	0.094
**Mal_M1**	0.336	0.004	0.380	0.486	0.003	0.455	0.351	0.003	0.396
**Mal_M2**	0.441	0.004	0.426	0.324	0.002	0.335	0.418	0.003	0.441
**Mal_M3**	0.110	0.001	0.097	0.043	0.001	0.040	0.096	0.001	0.068
**Mal_M4**	0.000	0.000	0.001	0.000	0.000	0.000	0.000	0.000	0.001
**Gln_M0**	0.023	0.001	0.041	0.023	0.001	0.080	0.031	0.001	0.040
**Gln_M1**	0.151	0.002	0.226	0.131	0.002	0.310	0.176	0.003	0.231
**Gln_M2**	0.461	0.006	0.422	0.575	0.013	0.408	0.466	0.006	0.438
**Gln_M3**	0.301	0.002	0.283	0.232	0.009	0.190	0.270	0.000	0.273
**Gln_M4**	0.063	0.003	0.029	0.039	0.002	0.011	0.058	0.002	0.018
**Gln_M5**	0.000	0.000	0.000	0.000	0.000	0.000	0.000	0.000	0.000
**Pro_M0**	0.087	0.001	0.041	0.112	0.002	0.080	0.093	0.002	0.040
**Pro_M1**	0.162	0.004	0.226	0.129	0.001	0.310	0.168	0.002	0.231
**Pro_M2**	0.425	0.006	0.422	0.519	0.007	0.408	0.459	0.004	0.438
**Pro_M3**	0.282	0.001	0.283	0.218	0.002	0.190	0.253	0.006	0.273
**Pro_M4**	0.045	0.001	0.029	0.022	0.002	0.011	0.028	0.002	0.018
**Pro_M5**	0.000	0.000	0.000	0.000	0.000	0.000	0.000	0.000	0.000

[a]M# refers to the numbers of the labelled carbon molecules in a metabolite.

[b]Simulated (sim) values are the respective MDVs of the OpenFLUX fitting yielding the lowest residual error.

Supplementary References

1. Cartharius, K. *et al.* MatInspector and beyond: Promoter analysis based on transcription factor binding sites. *Bioinformatics* **21,** 2933–2942 (2005).
2. Baumann, K. *et al.* A multi-level study of recombinant *Pichia pastoris* in different oxygen conditions. *BMC Syst. Biol.* **4,** 141 (2010).
