# Supplementary material for: A single Gal4-like transcription factor activates the Crabtree effect in Komagataella phaffii
Source: Nat Commun. 2018 Nov 21;9:4911. doi: 10.1038/s41467-018-07430-4 (PMC6249229; doi:10.1038/s41467-018-07430-4)
Supplement: Supplementary file 3 — Description of Additional Supplementary Files [file 41467_2018_7430_MOESM3_ESM.pdf]

Description of Additional Supplementary Files

File Name: Supplementary Data 1
Description: Transcriptome data of control, *CRA1* overexpression and *Δcra1* strains.
